# Oligomeric rearrangement may regulate channel activity

**DOI:** 10.52601/bpr.2023.230018

**Published:** 2024-10-31

**Authors:** Yue Ren, Xue Yang, Yuequan Shen

**Affiliations:** 1 State Key Laboratory of Medicinal Chemical Biology and Frontiers Science Center for Cell Responses, College of Life Sciences, Nankai University, Tianjin 300350, China

**Keywords:** Channel, Gating, Oligomeric rearrangement, CALHM, TRP channel

## Abstract

Channels are typically gated by several factors, including voltage, ligand and mechanical force. Most members of the calcium homeostasis modulator (CALHM) protein family, large-pore ATP release channels, exist in different oligomeric states. Dynamic conversions between CALHM1 heptamers and octamers to gate the channel were proposed. Meanwhile, the latest study observed that the transient receptor potential vanilloid 3 (TRPV3) channel adopts a dynamic transition between pentamers and canonical tetramers in response to small molecule treatment. These results suggest that oligomeric rearrangement may add a new layer to regulate the channel activities.

## INTRODUCTION

Channels play a vital role in a variety of physiological processes, which can be mainly divided into voltage-dependent gating, ligand gating and mechanical force gating. Voltage-dependent channels respond to transmembrane voltage gradients, causing changes in the conformation of the voltage-sensing domain, which rearrange the pore domain to open the channel (Tombola *et al.* 2006). The conformational change models of voltage-dependent channels include the helical screw, the paddle, the oscillating gate, and the interaction with membrane lipids (Kariev and Green 2012). Ligand-gated channels bind ligands to selectively permeate specific ions through the pore (Hucho and Weise 2001). Mechanosensitive channels convert mechanical disturbances into electrical or chemical signals, and the gating models include dragging, membrane dome formation, and tethering (Kefauver *et al.* 2020). Other gating mediators also include pH, light and redox potential changes (Woolley and Lougheed 2003), however, oligomeric rearrangements have not been previously reported.

## OLIGOMERIC CHANGE IN THE CALHM1 CHANNEL

The calcium homeostasis modulator (CALHM) protein family has six homologs (CALHM1-6) in vertebrates with 20%–50% sequence similarity (Foskett 2020; Taruno *et al.* 2013). CALHM1 is a voltage- and extracellular Ca^2+^-gated ATP release channel, which has been implicated in neural signaling and even Alzheimer's disease (Dreses-Werringloer *et al.* 2008; Taruno 2018). CALHM1 can also form heterologous complexes with CALHM3, which cannot generate conductance alone, to form fast voltage-gated ATP release channels in type II taste bud cells for downstream G-protein-coupled receptor-mediated taste perception (Ma *et al.* 2018). CALHM2, which can form undecamer hemichannels and gap junctions (Choi *et al.* 2019), mediates ATP release in astrocytes and regulates inflammatory activation in microglia (Cheng *et al.* 2021). CALHM4 gap junction channel and CALHM6 hemichannel, both of which can form two oligomeric states (10-mer and 11-mer), are abundantly expressed in placental tissues and have no significant voltage- or calcium-gated channel activity (Drozdzyk *et al.* 2020), but also participate in some physiological processes. CALHM6 is the only one in the family that is highly expressed in immune cells and regulates natural killer (NK) cell activation kinetics (Danielli *et al.* 2023). The function of CALHM5, which can form undecameric hemichannels (Bhat *et al.* 2021), needs to be further investigated.

**Figure 1:**
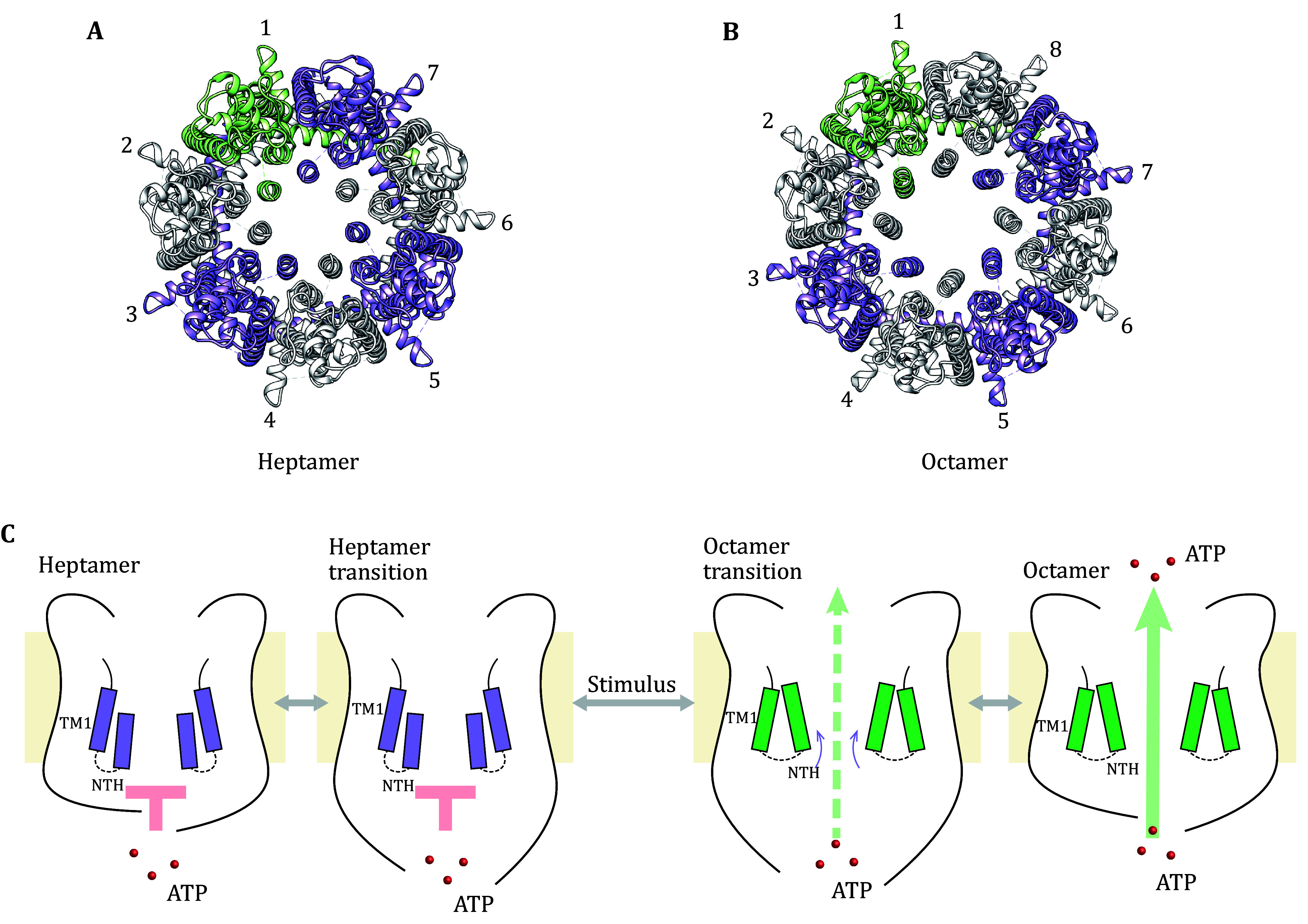
Putative regulatory mechanism of CALHM1 channel by oligomer rearrangement. **A** Structure of drCALHM1 heptamer (PDB 7DSE). **B** Structure of drCALHM1 octamer (PDB 6LYG). **C** Schematic drawing of oligomeric rearrangement regulation mechanism. In the resting state, the NTH of the heptameric CALHM1 is in the downward position. The pore is too narrow to pass through ATP molecules. When stimulated, NTH moves upward to widen the pore, triggering the formation of octamers. The pore is wide enough to permeate ATP molecules. The contours of two opposing monomers are shown as solid black lines. The yellow background represents the plasma membrane. TM1 and NTH of heptamers and octamers are shown in purple and green, respectively. Red spheres represent ATP molecules

In recent years, structures of the CALHM family, except CALHM3, have been resolved. The oligomeric states of CALHM family members vary, and even the same channel protein can exhibit different oligomeric states. In addition, cryo-EM structure studies of human connexin36 junction or hemichannel have been reported to have both 6-fold and 7-fold symmetry (Lee *et al.* 2023). At present, the putative regulatory mechanisms of CALHMs mainly include the transmembrane helix 1 (TM1) and the N-terminal helix (NTH) movement (Choi *et al.* 2019; Syrjanen *et al.* 2023), and lipids in the central pore and between helices (Syrjanen *et al.* 2020), but the differences in oligomerization states are not well explained. Ren *et al*. first reported the octameric structure of *Danio rerio* CALHM1 (drCALHM1) with a pore diameter of approximately 20 Å (Ren *et al.* 2020) (Fig. 1A). Later they discovered the existence of heptameric drCALHM1 with pore diameter of about 6.6 Å (Fig. 1B), and proposed the channel gating mechanism by oligomer states rearrangement for the first time (Ren *et al.* 2022). The main difference between the two oligomeric states lies in the position of the N-terminal helix (NTH). Molecular dynamics simulations indicate that the upward movement of NTH toward the extracellular space can lead to the deformation of the heptamer structure and a significant increase in the pore size. In the resting state, CALHM1 may exhibit a heptameric structure with NTH down. Upon sensory stimulation, the NTH moves upward toward the extracellular space, driving conformational changes, and oligomeric rearrangement to form the octamer, allowing the permeation of ATP molecules (Fig. 1C).

## OLIGOMERIC CHANGE IN THE TRPV3 CHANNEL

This way of regulating channel activity through oligomeric changes appears to be novel, introducing greater complexity to channel gating. Besides CALHM1 channels, does a similar regulation mechanism exist in other channels? Recently, Lansky *et al*. reported on the pentameric transient receptor potential vanilloid 3 (TRPV3), which differs from the previously established canonical tetramer (Lansky *et al.* 2023). TRPV3, a member of the TRP ion channel family involved in various physiological processes such as sensory function (Huffer *et al.* 2020), is primarily associated with skin temperature perception, wound healing, immune response, cancer, and other diseases (Su *et al.* 2023). Furthermore, they employed high-speed atomic force microscopy (HS-AFM) to observe the dynamic changes in the lipid bilayer, revealing the transient and reversible protomer exchange between pentamers and tetramers facilitated by membrane diffusion. They also identified that a chemical compound diphenylboronic anhydride (DPBA), can increase the proportions of TRPV3 pentamers. The cryo-EM structure of the TRPV3 pentamer indeed shows a dilated channel pore.

## SUMMARY AND PERSPECTIVES

The observation that oligomer rearrangement effectively regulates channel activity in two distinct channel types marks a significant breakthrough. This finding not only opens new pathways for gaining a deeper insight into channel mechanisms but also holds promise for innovative approaches in developing more effective drugs for diseases associated with channel dysfunction.

During the process of protomer exchange, a multitude of intriguing questions emerge, ranging from the necessity of an energy supply to the sources of this energy, the potential roles played by surrounding phospholipids, and the potential involvement of other auxiliary factors. These inquiries present compelling opportunities for further exploration and investigation.

As we witness advancements in technologies such as cryo-ET and expansion microscopy, there is a growing anticipation that similar regulatory mechanisms, centered around oligomer rearrangement, may soon be unveiled in other channels. This could herald a new era in our understanding of channel functions and provide novel avenues for tackling diseases linked to channel malfunction.

## Conflict of interest

Yue Ren, Xue Yang and Yuequan Shen declare that they have no conflict of interest.
